# Short- and long-term outcomes after heart transplantation in cardiac sarcoidosis and giant-cell myocarditis: a systematic review and meta-analysis

**DOI:** 10.1007/s00392-021-01920-0

**Published:** 2021-08-17

**Authors:** Emanuele Bobbio, Marie Björkenstam, Bright I. Nwaru, Francesco Giallauria, Eva Hessman, Niklas Bergh, Christian L. Polte, Jukka Lehtonen, Kristjan Karason, Entela Bollano

**Affiliations:** 1 Department of Cardiology, Sahlgrenska University Hospital, Gothenburg, Sweden; 2 Institute of Medicine At Sahlgrenska Academy, University of Gothenburg, Gothenburg, Sweden; 3 Krefting Research Centre, Institute of Medicine, University of Gothenburg, Gothenburg, Sweden; 4 Wallenberg Centre for Molecular and Translational Medicine, University of Gothenburg, Gothenburg, Sweden; 5 Department of Translational Medical Sciences, ‘Federico II’ University of Naples, Naples, Italy; 6 Biomedical Library, Gothenburg University Library, University of Gothenburg, Gothenburg, Sweden; 7 Departments of Clinical Physiology and Radiology, Sahlgrenska University Hospital, Gothenburg, Sweden; 8 Heart and Lung Centre, Helsinki University and Helsinki University Hospital, Helsinki, Finland; 9 Transplant Institute, Sahlgrenska University Hospital, Gothenburg, Sweden

**Keywords:** Inflammatory cardiomyopathy, Cardiac sarcoidosis, Giant-cell myocarditis, Heart transplantation, Meta-analysis, Systematic review

## Abstract

Heart transplantation (HTx) is a valid therapeutic option for end-stage heart failure secondary to cardiac sarcoidosis (CS) or giant-cell myocarditis (GCM). However, post-HTx outcomes in patients with inflammatory cardiomyopathy (ICM) have been poorly investigated. We searched PubMed, Scopus, Science Citation Index, EMBASE, and Google Scholar, screened the gray literature, and contacted experts in the field. We included studies comparing post-HTx survival, acute cellular rejection, and disease recurrence in patients with and without ICM. Data were synthesized by a random‐effects meta‐analysis. We screened 11,933 articles, of which 14 were considered eligible. In a pooled analysis, post-HTx survival was higher in CS than non-CS patients after 1 year (risk ratio [RR] 0.88, 95% confidence interval [CI] 0.60–1.17; *I*^2^ = 0%) and 5 years (RR 0.72, 95% CI 0.52–0.91; *I*^2^ = 0%), but statistically significant only after 5 years. During the first-year post-HTx, the risk of acute cellular rejection was similar for patients with and without CS, but after 5 years, it was lower in those with CS (RR 0.38, 95% CI 0.03–0.72; *I*^2^ = 0%). No difference in post-HTx survival was observed between patients with and without GCM after 1 year (RR 1.16, 95% CI 0.05–2.28; *I*^2^ = 0%) or 5 years (RR 0.98, 95% CI 0.42–1.54; *I*^2^ = 0%). During post-HTx follow-up, recurrence of CS and GCM occurred in 5% and 8% of patients, respectively. Post-HTx outcomes in patients with CS and GCM are comparable with cardiac recipients with other heart failure etiologies. Patients with ICM should not be disqualified from HTx.

**Graphic abstract:**

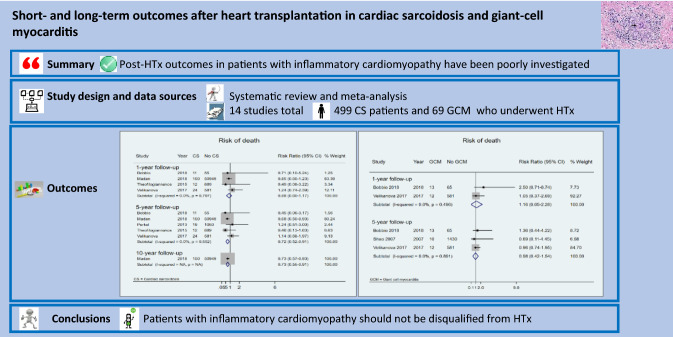

## Background

Inflammatory cardiomyopathy refers to a diverse group of disorders characterized by impaired cardiac function secondary to inflammation of the heart muscle [1]. A wide variety of infectious agents (most often viruses), systemic inflammatory diseases (including sarcoidosis, giant-cell myocarditis [GCM], and eosinophilic myocarditis), as well as hypersensitivity to certain toxic substances can be the underlying cause of this inflammation [2]. The natural history of inflammatory cardiomyopathy is highly variable and clinical features vary from mild symptoms to life-threatening arrhythmias and congestive heart failure (HF) which may require heart transplantation (HTx) [1, 2].

Sarcoidosis is a multisystem inflammatory disease of unknown etiology characterized by the presence of mononuclear phagocytes and non-caseating granulomas in different organ systems [3]. Although clinical heart disease has been confirmed in ≈5% of patients with systemic sarcoidosis, up to 25% of such patients display signs of cardiac sarcoidosis (CS) at autopsy indicating asymptomatic cardiac disease [4, 5]. Although HTx has been undertaken in a few CS patients with advanced HF or intractable arrhythmias [6, 7], with satisfactory short-term outcomes [6, 8, 9], little is known about long-term morbidity and mortality in this patient group [5, 10].

GCM is a rare fulminant heart disease that shares some clinical and histological features with CS, but is more aggressive [11, 12]. HTx has been the only definitive treatment for advanced GCM, but small studies have produced conflicting data regarding post-HTx outcomes [13–15]. In recent years, a small proportion of these patient may survive without HTx due to improvements in diagnostics and aggressive of immunosuppressive treatments. [13, 16]

The aim of this study was to collate information from single-center and registry studies to perform a systematic review and meta-analysis of post-HTx outcomes in patients with CS and GCM and compare them with those for transplant recipients with other HF etiologies.

## Methods

### Protocol registration and publication

The study protocol was developed in accordance with the recommendations of the preferred reporting items for systematic review and meta-analysis protocols (PRISMA-P) [17]. It was subsequently registered with PROSPERO (registration number: CRD42019140574) and published before undertaking the actual systematic review [18].

### Ethics

Ethical approval or informed consent was not required for this systematic review, because it was based only on previously published data, and did not involve any direct contact with individual patients.

### Eligibility criteria

Studies and conference abstracts reporting data on clinical outcomes (survival, acute cellular rejection, and disease recurrence) of patients who underwent HTx due to either CS or GCM were eligible for inclusion.

Given that our clinical question was prognostic, observational research was the most appropriate source including cross-sectional, case–control, and cohort studies. However, we also considered interventional or population-based studies embracing randomized-controlled trials, community studies, or field research. Only data on adult cardiac recipients aged ≥ 18 years were included. Sources that did not allow calculation of rates of the outcomes were excluded. There were no restrictions on language, date, or status of publication.

### Literature search strategy and information sources

We conducted the systematic review according to the PRISMA guidelines [19]. All keywords and commonly used terms referring to *cardiac sarcoidosis*, *giant-cell myocarditis*, and *heart transplantation*, in addition to Medical Subject Headings (MeSH) terms, were used. The full search strategies can be found in **SI 1** in the **Supplements.** We systematically searched electronic databases (PubMed, Scopus, Science Citation Index, and EMBASE) from their inception dates until the end of December 2019. Additional searches were conducted in Google Scholar in June 2019 and January 2020, but only the first 200 results were screened each time. Two authors (Em.B. and M.B.) independently screened the titles and/or abstracts of all retrieved articles for eligibility, after which the full texts of potentially eligible articles were reviewed. Any disagreement during the screening was resolved by group discussion. Additional articles were identified through review of different types of gray literature, conference abstracts, and trial registries, contact with researchers and communication between co-authors. A manual search of reference lists from the included studies was also performed, and appropriate references were evaluated using the same inclusion and exclusion criteria.

### Data extraction

A data extraction form was developed and, after being pilot-tested on five randomly selected studies, the template was refined and extended before being used for full data extraction of the included studies. Data were extracted by two authors (Em.B and M.B.) and independently checked for accuracy by a third reviewer (En.B.). Primary outcomes were defined as 1-, 5-, and 10-year mortality post-HTx. Secondary outcomes included acute cellular rejection and disease recurrence. The most comprehensively adjusted or, when unavailable, unadjusted risk ratio (RR), hazard ratio (HR), and 95% confidence intervals (CIs) were extracted or, when unavailable, calculated for each study. Seven authors were contacted for further information. Two replied, providing numerical data that had been presented graphically in the published work [20, 21]. Moreover, one of our co-authors (J.L.) provided additional unpublished data [21].

When studies with overlapping data were identified, only the publication with the largest number of patients was included in the meta-analysis. All serial publications for a particular cohort were nevertheless registered and tabulated. Any discrepancies were resolved by discussion with the contributing statistician (B.N.).

### Risk of bias in included studies

The quality of the studies was assessed by two reviewers (Em.B. and En.B.) using the Newcastle–Ottawa quality assessment scale (NOS) [22]. This tool contains three domains that assess the risk of bias, which may arise from the selection of a study group, comparability of study groups, and ascertainment of exposure or outcome. In case of discrepancies between reviewers, a consensus was reached after discussion among a broader group of co-authors. Three studies were not assessed for the risk of bias, since they were only available in abstract form and our contact with the authors was unsuccessful. [23–25]

### Statistical analysis

The meta-analysis was undertaken using Stata Statistical Software: Release 15 (StataCorp LP, College Station, TX). We employed random-effects meta-analysis to quantify the pooled effect estimates for studies considered to be sufficiently clinically, methodologically, and statistically homogeneous. We quantified the level of heterogeneity between studies using the *I*^2^ statistic accompanied by the P value indicating its statistical significance. The *I*^2^ statistic is a measure of estimating the percentage of variability in effect estimates due to heterogeneity rather than chance. The meta-analysis results are presented graphically by means of forest plots. In the meta-analysis, estimates from all studies were presented as RRs, with the exception of those in the study of Madan et al. [23], which were reported as HRs. We converted the HRs to (approximate) RRs using the recently proposed formulae provided by VanderWeele et al.[26]

## Results

### Literature search

**Fig. 1:**
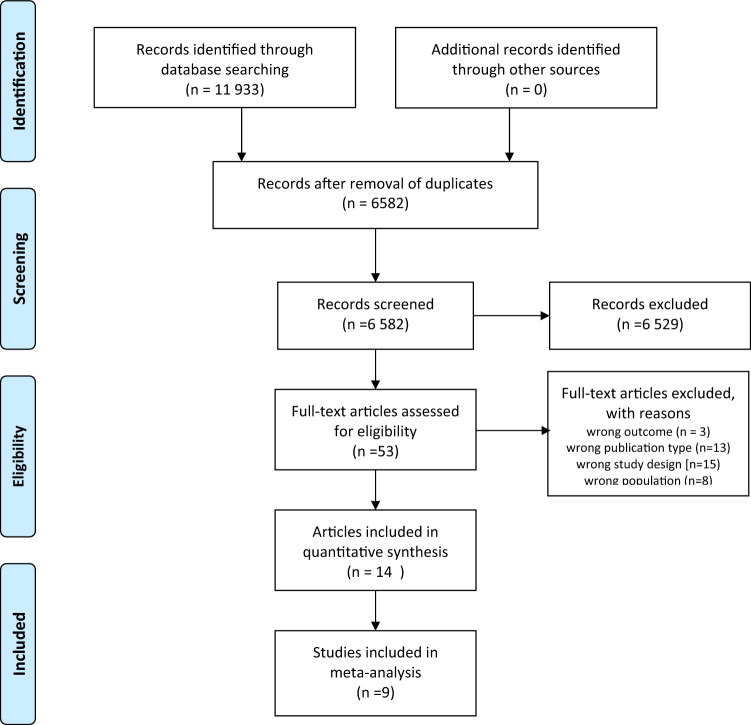
PRISMA flow diagram of studies on the outcomes of patients who underwent HTx because of either CS or GCM

The PRISMA flowchart depicting the 11,933 articles acquired by our search is shown in Fig. 1. After removal of duplicates, 6582 articles remained: 6529 were discarded after review of titles and/or abstracts and 39 of the remaining 53 were rejected after full-text review. Among the 14 remaining studies, there were published 9 articles [6–8, 14, 20, 27–30] and 5 peer-reviewed abstracts [21, 23–25, 31]. Two authors of peer-reviewed conference abstracts provided additional data, thus allowing for a more extensive assessment of their work [21, 31]. Altogether, the eligible studies reported on 499 CS, 69 GCM, 145,891 non-CS, and 16,297 non-GCM patients. Overall, nine papers were included in at least one meta-analysis. Considering that six studies investigated the same population with CS from the United Network for Organ Sharing (UNOS) database [6, 7, 23, 25, 27, 29], only two of these which included the largest number of patients reporting on the outcome of interest were included [23, 27].

### Study characteristics

The features of the 14 cohort studies included in this meta-analysis are presented in Tables 1 and 2. Ten observational studies were conducted in North America (72%), three in Europe (21%), and one in East Asia (7%). Ten studies reported separate outcomes for CS patients, two reported separate outcomes of patients with GCM, and two reported outcomes for both diseases. The overall 1-, 5-, or 10-year survival rates for patients with either CS [6–8, 20, 21, 23, 25, 27, 30, 31] or GCM [14, 21, 24, 31] after HTx were described in 12 publications. One- or 5-year acute cellular rejection rates after HTx for either CS [8, 21, 27, 30, 31] or GCM [14, 21, 24, 31], as well as disease recurrence at any time post-HTx in patients with CS [6, 8, 20, 21, 28, 30, 31] or GCM [21, 31], were reported in seven studies each. Histopathological diagnosis of acute cellular rejection in endomyocardial biopsies was reported according to the ISHLT grading scale [32, 33]. Most studies reported acute cellular rejections as grade ≥ 2R [6, 20, 21, 30, 31] or as freedom from any treated rejection [8]. A few papers reported cellular rejection as grade ≥ 1R [30] or as an unspecified grade of rejection [14, 24] and one paper described freedom from primary graft failure [27]. The presence of preoperative extra-cardiac sarcoidosis was described in 6 publications (Table 2) [6, 8, 20, 28, 30, 31]. Of a total of 73 CS patients included in these studies, 32 (43%) had known extra-cardiac organ involvement, 22 of which (30%) were diagnosed with pre-existing pulmonary sarcoidosis. Data on the immunosuppressive regimen applied during follow-up for the respective population in each publication are shown in Table 2.

**Table 1:** Main characteristics, key results, and overall quality of studies on the outcomes of patients who underwent HTx due to either CS or GCM

Reference, country; and study design	Study population size; etiology		Transplant period	Outcomes and assessment	Key results	Overall quality^c^
	Patients	Controls				
Akashi et al.[6] 2012; USA; retrospective cohort study, UNOS study^b^	14; CS	811; non-CS	1997–2010	Survival at 1 and 5 years post-HTx; recurrence within 3 years after HTx; cellular rejection ≥ grade 2 ISHLT	The clinical outcome of CS patients showed higher mortality than that of non-CS patients (1- and 5-year survival: 78.5% vs 87.2% and 52.4% vs 76.2%, respectively; *p* = 0.09). Only 2/14 cases showed recurrence of CS. Cellular rejection was observed in three patients within 6 months, and in 1 patient 18 months post-HTx. No data about rejection in the non-CS group is reported	Moderate
Bobbio et al.[27] 2019; Sweden; retrospective cohort study, single center^a^	13; GCM 11; CS	65; non-GCM 55; non-CS	1993–2018	Survival at 1 and 5 years post-HTx; recurrence any time after HTx; cellular rejection ≥ grade 2 ISHLT during the first year after HTx	Patients with either CS or GCM have similar post-HTx survival as HTx recipients treated due to other etiologies. Recurrence of GCM and CS was observed in 15% and 18% of patients, respectively. No difference in rejection rate was found	Moderate
Chang et al. [30] 2012; Taiwan; retrospective cohort study, single center^b^	5; CS	506; non-CS	1987–2010	Survival at 1 and 5 years post-HTx; recurrence within 3 years after HTx	All patients with CS were alive at 1- and 5-year follow-up; no data about non-CS group. No recurrence was reported	Moderate
Crawford et al. [24] 2018; USA; retrospective cohort study, UNOS study	67; CS	18,281; non-CS	2006–2015	Survival at 1 and 5 years post-HTx; freedom from primary graft failure at 5 years post-HTx	Compared with non-CS patients, CS patients had similar 1-year (91% vs 90%; log-rank *p* = 0.88) and 5-year (83% vs 77%; log-rank p = 0.46) freedom from mortality. Similar freedom from primary graft failure at 5 years was noted between CS and non-CS patients (93% and 92%, respectively; log-rank p = 0.76)	Moderate
DePasquale et al. [25] 2016; USA; retrospective cohort study, UNOS study^b^	102; CS	43,213; non-CS	1987–2013	Survival at 1, 5, and 10 years post-HTx	Crude 1-, 5-, and 10-year post-HTx survival was: CS patients (90%, 83%, and 51%, respectively); non-CS patients (86%, 71%, and 51%, respectively) (log-rank *p* = NS). Unadjusted HR for all-cause mortality post-HTx was 0.73 (CI 0.49–1.10). After adjustment (for age, sex, race, diabetes, ischemic time, dialysis, life support, waiting time, and HLA mismatch), HR was 0.63 (CI 0.38–1.05)	NA
DePasquale et al. [26] 2012; USA; retrospective cohort study, UNOS study^b^	81; CS	30,109; non-CS	1987–2010	Survival at 1, 5, and 10 years post-HTx	Data about 1-, 5-, and 10-year post-HTx survival is not shown for the CS subgroup	Moderate
Elamm et al. [14] 2017; USA; retrospective cohort study, UNOS study	32; GCM	14,221; IDCMP	1994–2015	Survival at 1, 5, and 10 years post-HTx; acute rejection during the index hospitalization; and rejection rates within 1 year post-HTx	The cumulative survivals for GCM patients at 1, 5, and 10 years were 94%, 82%, and 68%, respectively, which was similar to those for the other etiologies (*p* = 0.11). GCM patients had increased risk of acute cellular rejection compared with IDCMP patients (16% vs 5%; p = 0.021) but no difference in re-hospitalization for rejection	Moderate
Madan et al. [22] 2017; USA; retrospective cohort study, UNOS study	150; CS	50,949; non-CS	1987–2015	Survival at 1, 5, and 10 years post-HTx	CS recipients had similar 1-year mortality [Cox HR 0.79 (CI 0.47–1.34), *p* = 0.390], but significantly lower 5-year [Cox HR 0.57 (CI 0.36–0.90), *p* = 0.015] and 10-year [Cox HR 0.63 (CI 0.44–0.90), p = 0.012] mortality post-HTx in comparison with non-CS recipients	NA
Perkel et al. [8] 2013; USA; retrospective cohort study, single center	19; CS	1,050; non-CS	1991–2010	Survival at 5 years post-HTx; recurrence of the disease; 1-year freedom from any treated rejection	There were no statistically significant differences between CS and non-CS patients in 1-year freedom from any treated rejection (79% vs 90%) and 5-year post-HTx survival (79% vs 83%). No patients had recurrence of sarcoidosis in the allograft	Moderate
Rosenthal et al. [28] 2018; USA; retrospective cohort study, single center	12; CS	28; non-CS	1995–2016	Post-HTx survival; any cellular rejection ≥ grade 1a ISHLT and cellular rejection ≥ grade 2 ISHLT	Patients with CS had excellent survival after HTx, with no deaths. Cellular rejection ≥ grade 1a ISHLT occurred less frequently in the CS group than in the non-CS group (17% and 68%, respectively; *p* = 0.006); and none of 12 patients in the CS group experienced ≥ 2 rejections	Moderate
Shao et al. [29] 2007; USA; retrospective cohort study, single center	14; GCM	1,430; non-GCM	1984–2004	Survival at 5 years post-HTx; any treated rejection at 5 years post-HTx	Five-year survival was similar between GCM and non-GCM patients (90% vs 85.5%; *p* = 0.73). There was no difference in any treated rejection (30% vs 26.2%; *p* = 0.73)	NA
Theofilogiannakos et al. [19] 2015; UK; retrospective cohort study, single center^a^	12; CS	889; non-CS	1990–2012	Survival at 1 and 5 years post-HTx; disease recurrence; cellular rejection grade ≥ 2 ISHLT during the first year after HTx	CS patients had excellent post-HTx outcomes with survival rates of 92% at 1 year and 83% at 5 years. Survival was similar to that of patients who underwent HTx due to other etiologies. No recurrence of CS was reported. No data about rejection in non-CS group is shown	Moderate
Velikanova et al. [20] 2017; Finland; retrospective cohort study, single center^a^	24; CS 12; GCM	581; non-CS 581; non-GCM	1987–2020	Survival at 1 and 5 years post-HTx; recurrence any time after HTx; cellular rejection > grade 2 ISHLT during the first year after HTx	Patients with either CS or GCM have similar post-HTx survival to that of HTx recipients treated due to other etiologies. Recurrence of GCM and CS was observed in 0% and 4% of patients, respectively. A statistically significant increased risk of cellular rejection was reported in the CS group compared with the non-CS group at 1-year follow-up. No differences in rejection rate were found between GCM and non-GCM patients at 1 year post-HTx	Moderate
Zaidi et al. [7] 2007; USA; retrospective cohort study, UNOS study^b^	65; CS	38,165; non-CS	1987–2007	Survival at 1 and 5 years post-HTx; cellular rejection > grade 2 ISHLT during the first year after HTX	One-year post-HTx survival was significantly better for CS compared with non-CS patients (87.7% vs 84.5%; *p* = 0.03). No significant differences were found up to 5 years after HTx when survival was compared by gender, UNOS status, and transplant era	Moderate

*CI* confidence interval; *CS* cardiac sarcoidosis; *GCM* giant cell myocarditis; *HLA* human leukocyte antigen; *HR* hazard ratio; *HTx* heart transplantation; *IDCMP* idiopathic dilated cardiomyopathy; *ISHLT* International Society for Heart and Lung Transplantation; *UNOS* United Network for Organ Sharing

^a^The authors provided additional unpublished data

^b^Study not included in any forest plot due to patient overlap or absence of events registered

^c^The risk of bias was not assessed in studies providing insufficient details

**Table 2:** Demographic characteristics and immunosuppression regimen of patients who underwent HTx due to either CS or GCM

Reference	Study population age (years)^a^	Study population gender (male, %)	Diagnosis	Extra-cardiac involvement	Immunosuppression regimen
Akashi et al. [6]	CS: 51 ± 9; non-CS: 53 ± 4 (*p* value = ns)	CS: 50%; non-CS 77.2% (*p* value = 0.0002)	Six patients were diagnosed with sarcoidosis before HTx with one patient diagnosed by EMB, one patient on myocardial core tissue at the time of VAD, and 4 patients diagnosed by pre-existing concomitant pulmonary sarcoidosis. The remaining 8 patients (57.1%) were diagnosed with CS at the time of HTx	Six patients (43%) received a diagnosis of pulmonary sarcoidosis before HTx	Following HTx, all patients received triple immunosuppression regimen using calcineurin inhibitors, mycophenolate, and steroids
Bobbio et al. [27]^b^	CS: 50.2 ± 12.5; non-CS: 50.1 ± 12.5 (*p* value = ns); GCM: 45.6 ± 13.3; non-GCM: 45.3 ± 13.7 (*p* value = ns)	Controls were matched by gender	EMB confirmed diagnosis in 4/11 (36%) patients with CS and 10/13 (77%) patients with GCM. The remaining were diagnosed by pathological investigation of the explanted heart	Four (36%) of CS received a diagnosis of extra-cardiac sarcoidosis before HTx	Following HTx, all patients received triple immunosuppression regimen using calcineurin inhibitors, mycophenolate, and steroids
Chang et al. [30]	CS: 34.9 ± 8; non-CS: 44 ± 16 (*p* value = ns)	CS: 80%; non-CS 71% (*p* value = NA)	Only one patient had documented sarcoidosis before HTx. The remaining were diagnosed by pathological investigation of the explanted heart	No one had extra-cardiac involvement	Anti-thymocyte globulin for induction and azathioprine 1 h before the operation with Solumedrol. Following HTx, all patients received triple immunosuppression regimen using calcineurin inhibitors, mycophenolate, and steroids
Crawford et al. [24]	CS: 51 (47–59); non-CS: 56 (46–62) (*p* value = ns)	NA	NA	NA	NA
DePasquale et al. [25]	NA	NA	NA	NA	NA
DePasquale et al. [26]	CS: 50 ± 9; non-CS: 52 ± 12 (*p* value = NA)	CS; 59%; non-CS: 78% (*p* value = NA)	NA	NA	NA
Elamm et al. [14]	GCM: 52 (40–55); IDCMP: 52 (43–59) (*p* value = ns)	GCM: 63%; IDCMP: 72% (*p* value = ns)	The authors used the UNOS organ transplantation files to identify patients with GCM by interrogating the primary diagnosis free text entry field	NA	Of the 32 patients with GCM, 11 underwent induction therapy. At the time of discharge from index hospitalization, 28 patients were on steroids, 20 on tacrolimus, 24 on mycophenolate, and 4 on azathioprine at the time of discharge. The most common regimen used was steroids + tacrolimus + mycophenolate in 16 patients, whereas 7 received steroid + cyclosporine + mycophenolate
Madan et al. [22]	CS: 51 (46–58); non-CS 54 (46–61) (*p* value = 0.02)	NA	NA	NA	NA
Perkel et al. [8]	CS ranged in age from 29 to 68 years; no data about non-CS group	CS: 53%; no data about non-CS group	Four patients (21%) had biopsy-confirmed cardiac sarcoidosis before HTx, and these patients, as well as the other 15 patients had CS confirmed with pathological examination of the explanted heart	Eight patients (42%) had known preoperative extra-cardiac sarcoidosis	Following HTx, all patients received triple immunosuppression regimen using calcineurin inhibitors, mycophenolate, and steroids
Rosenthal et al. [28]	CS: 58.6; non-CS: 56.2 (*p* value = ns)	CS: 58%; non-CS: 18% (*p* value = 0.02)	All patients had CS diagnosis before HTx. Diagnosis was made either preoperatively using Heart Rhythm Society expert consensus criteria or confirmed by histological findings	Nine patients (75%) had known preoperative extra-cardiac sarcoidosis	Induction with anti-thymocyte globulin. Following HTx, all patients received triple immunosuppression regimen using calcineurin inhibitors, mycophenolate, and steroids
Shao et al. [29]	NA	NA	NA	NA	NA
Theofilogiannakos et al. [19]^b^	CS: 41.2 ± 10; non-CS: 48.5 ± 11.3 (*p* value = 0.026)	CS: 75%; non-CS: 78% (*p* value = ns)	Pre-HTx diagnosis was established in 4 patients due to lung and cutaneous involvement. In the remaining 8 patients, CS was only diagnosed by pathological examination of the explanted heart	Five patients (42%) had known preoperative extra-cardiac sarcoidosis	Following HTx, all patients received triple immunosuppression regimen using calcineurin inhibitors, mycophenolate, and steroids
Velikanova et al. [20]^b^	NA	NA	CS diagnosis was confirmed with pathological examination of the explanted heart in all patients	NA	Following HTx, all patients received triple immunosuppression regimen using calcineurin inhibitors, mycophenolate, and steroids
Zaidi et al. [7]	CS: 46 (0–77); non-CS: 45.5 (2–63) (*p* value = NA)	CS: 61.5%; non-CS: 75.5% (*p* value = 0.009)	NA	NA	NA

*CS* cardiac sarcoidosis; *EMB* endomyocardial biopsy; *GCM* giant cell myocarditis; *HTx* heart transplantation; *IDCMP* idiopathic dilated cardiomyopathy; *VAD* ventricular assist device; *UNOS* United Network for Organ Sharing

^a^Data reported as mean ± SD or median (CI)

^b^The authors provided additional unpublished data

### Reason for HTx

The meta-analysis did not provide granular information on what grounds patients with CS and GCM were listed for HTx. Still, according to the literature and in line with our own experience, chronic end-stage HF caused by CS may develop slowly, often during several years, with or without atrioventricular block and/or ventricular arrhythmias. GCM, on the other hand, is characterized by acute fulminant heart failure that develops within a few days or weeks, frequently associated with treatment-resistant ventricular arrhythmias, which demands rapid diagnosis and intervention.

### Risk of bias within studies

Overall quality based on the grading according to the NOS [22] was found to be moderate for all 11 studies that provided sufficient details to assess the risk of bias (Table 3). Ratings were lowest in the domains ‘Outcome assessment’ and ‘Confounding’.

**Table 3:** Domain-specific quality assessment of studies on post-HTx outcomes in patients with either CS or GCM

Reference; country	Overall quality	Component quality				
		Study design	Exposure assessment	Outcome assessment	Sample size	Confounding
Akashi et al. [6]; USA	Moderate	High	High	Moderate	Moderate	Low
Bobbio et al. [31] 2019; Sweden	Moderate	Moderate	High	Moderate	Moderate	Moderate
Chang et al. [28] 2012; Taiwan	Moderate	Moderate	High	Low	Moderate	Low
Crawford et al. [24] 2018; USA	Moderate	High	High	Moderate	High	Low
DePasquale et al. [26] 2012; USA	Moderate	Moderate	High	Low	High	Low
Elamm et al. [14] 2017; USA	Moderate	Moderate	High	Low	Moderate	Low
Perkel et al. [8] 2013; USA	Moderate	Moderate	High	Moderate	Moderate	Low
Rosenthal et al. [28] 2018; USA	Moderate	Moderate	Moderate	High	Moderate	Moderate
Theofilogiannakos et al. [19] 2015; UK	Moderate	High	High	Low	Moderate	Low
Velikanova et al. [20] 2017; Finland	Moderate	Moderate	High	Moderate	Moderate	Low
Zaidi et al. [7] 2007; USA	Moderate	High	High	Low	High	Low

The risk of bias was not assessed in three studies providing insufficient details [22, 25, 29]

### Post-transplant survival

**Fig. 2:**
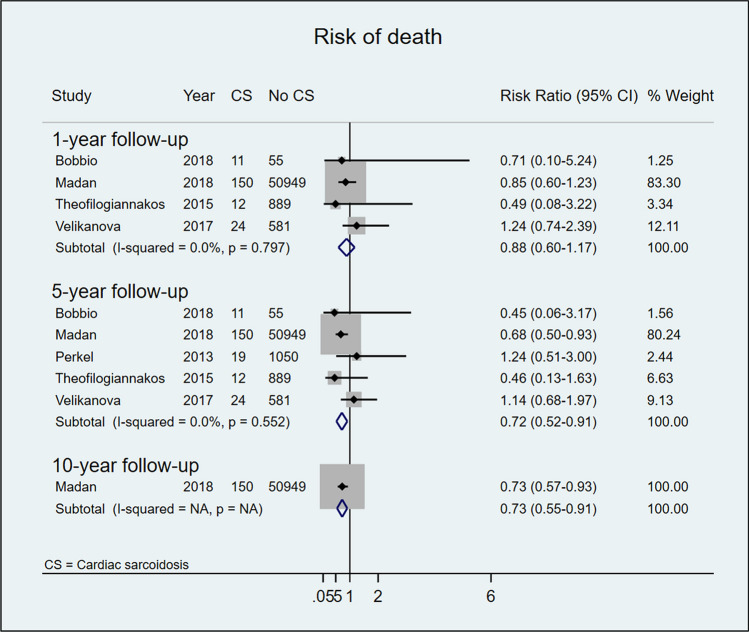
Risk of post-HTx death in patients with and without CS after 1, 5, and 10 years of follow-up

Patients who underwent HTx due to CS displayed higher 1-, 5-, and 10-year survival rates than non-CS comparators (85% vs 83% at 1 year and 82% vs 76% at 5 years post-HTx). However, while the pooled results at 1-year follow-up did not achieve statistical significance (RR 0.88, 95%CI 0.60–1.17; I^2^ = 0%, p value for I^2^ = 0.797), the results for survival at 5-year follow-up did (RR 0.72, 95%CI 0.52–0.91; I^2^ = 0%, p value for I^2^ = 0.552). Only one study reported survival at 10-year post-HTx follow-up and found a significantly higher survival rate among patient with CS than among controls (RR 0.73, 95%CI 0.55–0.91) (Fig. 2).

**Fig. 3:**
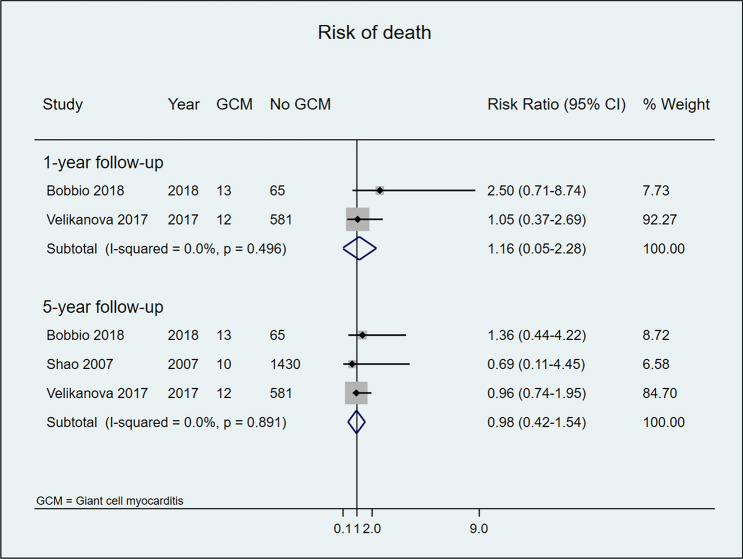
Risk of post-HTx death in patients with and without GCM after 1 and 5 years of follow-up

No statistically significant difference in post-HTx survival was observed between patients with or without GCM at 1- or 5-year follow-up (80% vs 85% and 82% vs 84%, respectively) (Fig. 3).

### Acute cellular rejection rate

**Fig. 4:**
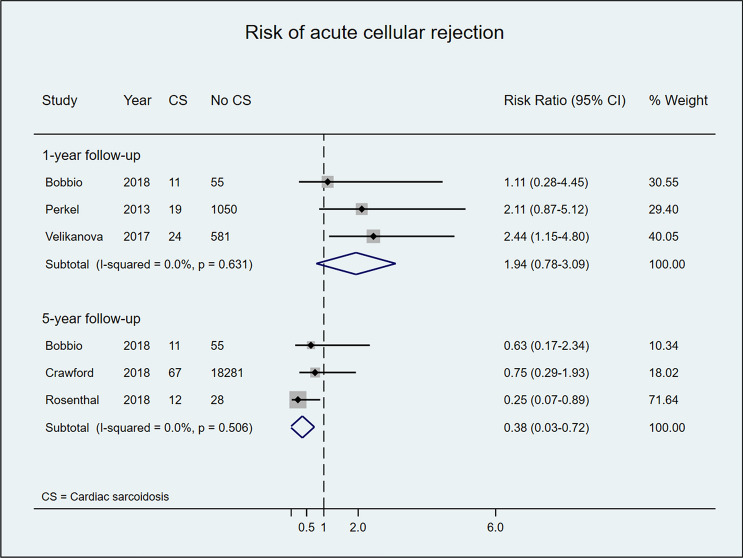
Risk of acute cellular rejection in patients who underwent HTx due to CS versus other HF etiologies after 1 and 5 years of follow-up

Acute cellular rejection rates in post-HTx CS patients are displayed in Fig. 4. Most included studies [8, 21, 31] reported a non-significantly higher risk for acute cellular rejection among patients with CS versus controls during the first-year post-HTx, a finding confirmed in the meta-analysis of pooled data (RR 1.94, 95% CI 0.78–3.09; *I*^2^ = 0%, p value for *I*^2^ = 0.631). In meta-analysis of pooled data after 5 years of follow-up, however, the risk of acute rejection was significantly lower in patients with CS than in controls (RR 0.38, 95%CI 0.03–0.72; I^2^ = 0%, *p* value for I^2^ = 0.506) (Fig. 4).

**Fig. 5:**
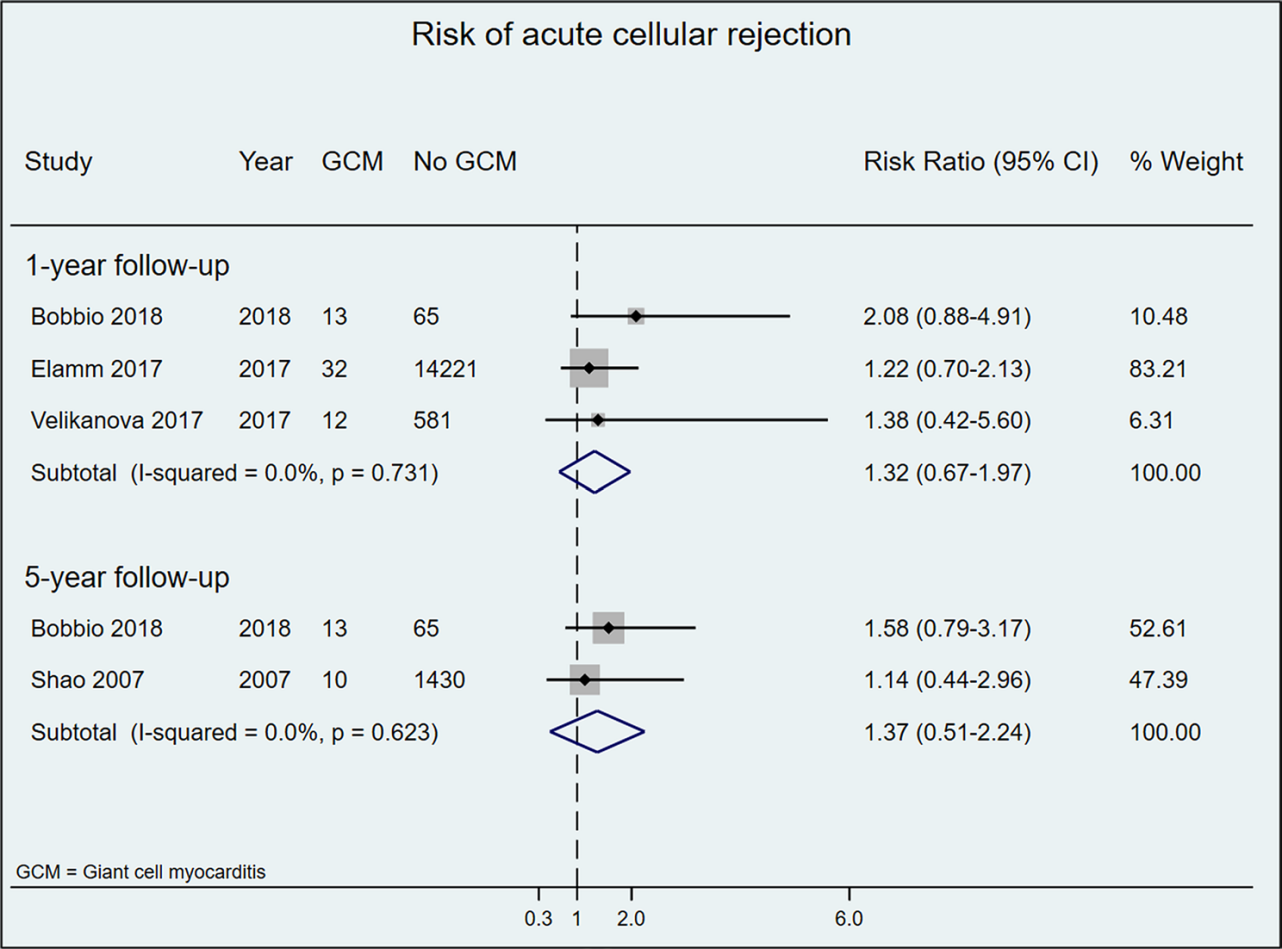
Risk of acute cellular rejection in patients who underwent HTx due to GCM versus other HF etiologies after 1 and 5 years of follow-up

Acute cellular rejection rates in post-HTx GCM patients, in both individual studies and meta-analysis, indicated non-significant increases in the risk of acute cellular rejection at 1- and 5-year follow-up (Fig. 5).

### Disease recurrence

The information provided on CS or GCM recurrence after HTx varied between studies and seven reports offered no data with respect to disease relapse [7, 14, 23–25, 27, 29]. In three studies, recurrence of CS was reported in 4% [21], 14% [6], and 18% [31] of HTx patients; and no disease recurrence was recorded in four other studies [8, 20, 28, 30]. GCM relapse after HTx was observed in 15% of patients in one study [31]; and no recurrence was reported in the study of Velikanova et al. [21]

## Discussion

The results of this systematic review and meta-analysis of 499 patients with CS and 69 with GCM who underwent HTx found that: (i) CS patients displayed a consistently better survival rate and similar risk of developing acute cellular rejection post-HTx compared with controls; (ii) no statistically significant difference was observed between post-HTx patients with or without GCM in terms of either outcome.

We developed, registered, and published a detailed protocol prior to undertaking the review [18], which enhanced the transparency of the review process. We undertook an extensive search of major medical and public health databases, supplemented by screening of the gray literature and contacting expert physicians in the field. It is highly unlikely that we missed any relevant literature.

In our meta-analysis, the CS group showed better post-HTx survival at 1- and 5-year follow-up after HTx, with statistically significant survival benefit at 5-year follow-up and in the one study that followed the patient population for ≥ 10 years [23]. Post-HTx CS patients showed a non-significantly higher risk for acute cellular rejection during the first year, but a substantially reduced risk for rejection compared with non-CS patients at 5-year follow-up. Although our findings indicate that CS patients appear to have a favorable outcome after HTx, appropriate diagnosis and careful patient selection are still essential. Thorough assessment of systemic involvement as well as a concerted effort to determine HF etiology during pre-transplant work-up are likely to be important for post-HTx outcome [34, 35]. Furthermore, most HTx centers have adopted a dedicated immunosuppression strategy for CS patients including induction therapy (with either thymoglobulin or basiliximab) and long-term treatment with low-dose prednisolone [36, 37].

Despite an advantageous outcome, some centers are still hesitant to transplant CS patients due to concerns about disease recurrence [36]. In the early post-transplant period, when higher doses of immunosuppressive agents are applied, it is expected that cardiac sarcoidosis will be quiescent. Although recurrence of CS was observed in recipients of solid organ transplantation following tailoring of medications to maintenance levels [38, 39], more recent studies have suggested that treatment of emerging cellular and/or humoral rejections could prevent CS reactivation after HTx [7, 30]. We found that around 5% of patients (range 0 − 18%) developed recurrence of sarcoidosis in the allograft any time post-HTx. Therefore, prolonged surveillance for CS relapse and a long-term immunosuppressive regimen including low-dose prednisolone should be considered to prevent disease relapse. Corticosteroids remain the cornerstone of treatment for sarcoidosis and, in our experience, recurrence of CS in the allograft easily resolves after steroid pulse therapy.

HTx is currently the best therapeutic option in patients with advanced GCM or when aggressive immunosuppressive treatment fails. However, increased risk of early rejection and disease recurrence have been reported and the prognosis of following HTx in GCM remains unclear [11, 13, 40]. Our analysis demonstrates that 1- and 5-year survival rates in GCM patients were similar to those in transplant recipients with other HF etiologies. All included studies showed a tendency toward an increased risk of acute cellular rejection in patients with GCM but no aggregate statistically significance difference versus controls. Similar results were reported by Elamm et al.[14] using data from the UNOS registry. Despite higher rates of rejection, 32 GCM patients displayed similar post-HTx survival when compared with 14,221 patients transplanted due to idiopathic dilated cardiomyopathy [14]. That study was excluded from our survival analysis, since the authors did not respond to our request for additional information.

GCM relapse may occur in the transplanted heart despite ongoing immunosuppressive treatment. In this study, around 8% (range 0 − 15%) of patients developed recurrence of giant cells in the allograft at any time after HTx. A standard immunosuppressive regimen including a calcineurin inhibitor, mycophenolate mofetil, and prednisolone is probably sufficient to prevent disease recurrence in patients transplanted due to GCM. According to experience from our own and other centers, caution should be exercised when tapering corticosteroid treatment [41, 42]. Moreover, treatment with anti-thymocyte globulin in the peri-transplant period has been suggested to limit the recurrence of GCM in a small cohort of seven patients in the early phase after HTx [43]. The overall survival rate and the favorable response to therapy identified in our meta-analysis suggest, however, that HTx in patients with GCM can be considered safe from a graft-survival perspective.

The present review and meta-analysis is the most comprehensive and robust synthesis of the evidence on this topic and addresses concerns about post-HTx outcome in inflammatory cardiomyopathies. Publication bias was minimized by performing a comprehensive literature search and contacting authors who have published in the field, through which we were able to identify additional studies, including conference abstracts [44]. Nevertheless, certain limitations of our work should be acknowledged. The potential for double counting of patients in the UNOS scheme has been noted. Given the small number of studies for the meta-analysis of each outcome, we could not evaluate the potential influences of publication bias or small-study effect on our results. Sample size limitations also prevented us from undertaking the pre-planned subgroup analyses on quality of study, country, age, gender, ethnicity, and transplant era, and also precluded meaningful sensitivity analyses. Furthermore, the studies had different sample sizes and contributed differently to the result of the pooled analysis, with larger trials, as expected having a larger contribution to the final estimate. However, there was no heterogeneity in the association between exposure and outcomes among the studies.

## Conclusion

Patients with CS treated with HTx appear to have consistently better short- and long-term survival rates and greater freedom from primary graft failure compared with cardiac recipients with other HF etiologies. Post-HTx survival was similar for patients with and without GCM. Neither CS nor GCM patients displayed a higher risk for acute cellular rejection than other transplant recipients. These data support the continued use of HTx for patients with inflammatory cardiomyopathies given correct diagnosis, appropriate patient selection, and adequate post-HTx management.

## Data Availability

Not applicable.

## Availability of data

The data that support the findings of this study are available from the corresponding author, Em.B., upon reasonable request.
